# 2-[(*E*)-(2-Hy­droxy­naphthalen-1-yl)methyl­idene­amino]­isoindoline-1,3-dione

**DOI:** 10.1107/S1600536811045569

**Published:** 2011-11-05

**Authors:** Hua-Jie Xu, Peng-Fei Su, Zhao-Di Liu

**Affiliations:** a Department of Chemistry, Fuyang Normal College, Fuyang Anhui 236041, People’s Republic of China; b Xi’an Modern Chemistry, Research Institute, Xi’an Shanxi 710065, People’s Republic of China

## Abstract

The title compound, C_19_H_12_N_2_O_3_, has two independent mol­ecules (*A* and *B*) in the asymmetric unit. There is an intra­molecular O—H⋯N hydrogen bond in each mol­ecule. The mean planes of the naphthalene [maximum deviations = 0.024 (3) and 0.030 (3) Å in *A* and *B*, respectively] and the isoindoline units [maximum deviations 0.009 (3) and 0.008 (3) Å in *A* and *B*, respectively] are almostly coplanar, with dihedral angles of 4.25 (9) ad 3.84 (9)° in mol­ecules *A* and *B*, respectively. The two independent mol­ecules are connected by π–π inter­actions [centroid-centroid distances 3.5527 (19) and 3.5627 (19) Å]. In the crystal, the *A*+*B* pairs are further connected via π–π inter­actions [centroid–centroid distances = 3.693 (2)–3.831 (2) Å], leading to the formation of columns propagating along the *a*-axis direction. The columns are linked *via* C—H⋯O inter­actions, leading to the formation of a three-dimensional network.

## Related literature

For details concerning the naphthalene group as a fluoro­phore and as a fluorescent chemosensor, see: Li *et al.* (2010 ▶); Liu *et al.* (2011 ▶); Iijima *et al.* (2010 ▶); Hosseini *et al.* (2010 ▶); Singh *et al.* (2008 ▶).
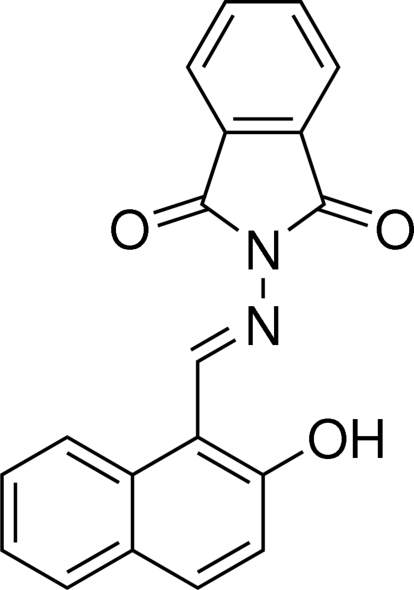


## Experimental

### 

#### Crystal data

C_19_H_12_N_2_O_3_
*M* *_r_* = 316.31
Monoclinic, 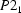

*a* = 7.153 (2) Å
*b* = 15.503 (4) Å
*c* = 13.446 (4) Å
β = 100.763 (5)°
*V* = 1464.7 (7) Å^3^
*Z* = 4
Mo *K*α radiation
μ = 0.10 mm^−1^
*T* = 298 K
0.20 × 0.20 × 0.10 mm

#### Data collection

Siemens SMART CCD area-detector diffractometer
Absorption correction: multi-scan (*SADABS*; Sheldrick, 1996 ▶) *T*_min_ = 0.981, *T*_max_ = 0.990
8507 measured reflections
5907 independent reflections
4475 reflections with *I* > 2σ(*I*)
*R*_int_ = 0.026

#### Refinement

*R*[*F* ^2^ > 2σ(*F* ^2^)] = 0.043
*wR*(*F* ^2^) = 0.109
*S* = 1.02
5907 reflections
433 parameters
1 restraint
H-atom parameters constrained
Δρ_max_ = 0.14 e Å^−3^
Δρ_min_ = −0.19 e Å^−3^
Absolute structure: Flack (1983 ▶), 2608 Friedel pairs
Flack parameter: 0.2 (12)

### 

Data collection: *SMART* (Siemens, 1996 ▶); cell refinement: *SAINT* (Siemens, 1996 ▶); data reduction: *SAINT*; program(s) used to solve structure: *SHELXS97* (Sheldrick, 2008 ▶); program(s) used to refine structure: *SHELXL97* (Sheldrick, 2008 ▶); molecular graphics: *SHELXTL* (Sheldrick, 2008 ▶); software used to prepare material for publication: *SHELXTL*.

## Supplementary Material

Crystal structure: contains datablock(s) global, I. DOI: 10.1107/S1600536811045569/su2339sup1.cif

Structure factors: contains datablock(s) I. DOI: 10.1107/S1600536811045569/su2339Isup2.hkl

Supplementary material file. DOI: 10.1107/S1600536811045569/su2339Isup3.cml

Additional supplementary materials:  crystallographic information; 3D view; checkCIF report

## Figures and Tables

**Table 1:** Hydrogen-bond geometry (Å, °)

*D*—H⋯*A*	*D*—H	H⋯*A*	*D*⋯*A*	*D*—H⋯*A*
O1—H1⋯N1	0.82	1.83	2.555 (3)	147
O4—H4*A*⋯N3	0.82	1.93	2.555 (3)	133
C18—H18⋯O6^i^	0.93	2.53	3.204 (4)	129
C35—H35⋯O4^ii^	0.93	2.56	3.339 (4)	141

Symmetry codes: (i) 
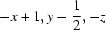
; (ii) 
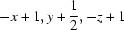
.

**Fig. 1.**
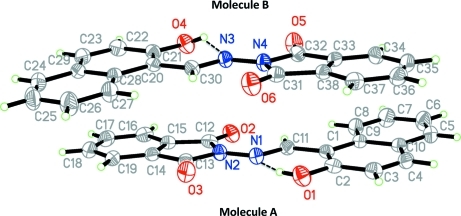
The molecular structure of the two independent molecules (A and B) of the title compound, showing 30% probability displacement ellipsoids and the atom numbering scheme. The intramolecular O-H···N hydrogen bonds are shown as dashed lines.

**Fig. 2.**
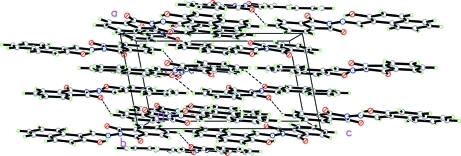
Crystal packing, viewed along the b axis, of the title compound. The C-H···O interactions are shown as dashed lines (see Table 1 for details).

## supplementary crystallographic information

### Comment

The naphthalene group as a fluorophore has been studied extensively due to its characteristic photophysical properties and the competitive stability in the environment (Li *et al.*, 2010; Liu *et al.*, 2011; Iijima *et al.*, 2010; Hosseini *et al.*, 2010; Singh *et al.*, 2008). As part of an ongoing study of such compounds based on the naphthalene group for fluorescent chemosensors (Liu *et al.*, 2011), we herein report on the crystal structure of the title compound.

The molecular structure of the two indepedent molecules (A and B) in the title compound are shown in Fig. 1. There is an intramolecular O-H···N hydrogen bond in each molecule (Table 1). The naphthalene [(C1-C10) in A and (C20-C28) in B] and the isoindoline [(N2,C12-C19) in A and (N4,C31-C38) in B] ring systems are almostly co-planar, with dihedral angles of 4.25 (9) ad 3.84 (9)° in molecules A and B, respectively. The A and B molecules are linked via π–π interactions, Cg2···Cg8 = 3.5627 (19) Å and Cg4···Cg6 = 3.5527 (19) Å (see Table 2 for details).

In the crystal further π–π interactions (Table 2), connect the pairs of A+B molecules to form columns propagating in the a-axis direction. There are also C-H···O interactions present that link the columns to form of a three-dimensional network (Table 1 and Fig. 2).

### Experimental

A solution of 2-aminoisoindoline-1,3-dione (0.16 g, 1 mmol) in 15 ml ethanol was added slowly to a solution containing 2-chlorobenzaldehyde (0.14 g,1 mmol) in 5 ml absolute ethanol under heating and stirring. The mixture was refluxed for 2 h, and then cooled to room temperature. The resulting solution was left to stand in air for 15 days. Colourless prism-shaped crystals were formed on slow evaporation of the solvent.

### Refinement

All H-atoms were placed in calculated positions and treated as riding: C—H = 0.93 Å, O—H = 0.82 Å, with *U*_iso_(H) = 1.2*U*_eq_(parent C-atom) and 1.5*U*_eq_(parent O-atom). The Flack parameter, 0.2 (12), has no meaning.

### Crystal data

**Table:**

C_19_H_12_N_2_O_3_	*F*(000) = 656
*M**_r_* = 316.31	*D*_x_ = 1.434 Mg m^−^^3^
Monoclinic, *P*2_1_	Mo *K*α radiation, λ = 0.71073 Å
Hall symbol: P 2yb	Cell parameters from 2910 reflections
*a* = 7.153 (2) Å	θ = 2.6–26.2°
*b* = 15.503 (4) Å	µ = 0.10 mm^−^^1^
*c* = 13.446 (4) Å	*T* = 298 K
β = 100.763 (5)°	Prism, yellow
*V* = 1464.7 (7) Å^3^	0.20 × 0.20 × 0.10 mm
*Z* = 4	

### Data collection

**Table:**

Siemens SMART CCD area-detector diffractometer	5907 independent reflections
Radiation source: fine-focus sealed tube	4475 reflections with *I* > 2σ(*I*)
graphite	*R*_int_ = 0.026
φ and ω scans	θ_max_ = 27.0°, θ_min_ = 1.5°
Absorption correction: multi-scan (*SADABS*; Sheldrick, 1996)	*h* = −8→9
*T*_min_ = 0.981, *T*_max_ = 0.990	*k* = −19→19
8507 measured reflections	*l* = −12→17

### Refinement

**Table:**

Refinement on *F*^2^	Secondary atom site location: difference Fourier map
Least-squares matrix: full	Hydrogen site location: inferred from neighbouring sites
*R*[*F*^2^ > 2σ(*F*^2^)] = 0.043	H-atom parameters constrained
*wR*(*F*^2^) = 0.109	*w* = 1/[σ^2^(*F*_o_^2^) + (0.0506*P*)^2^ + 0.109*P*] where *P* = (*F*_o_^2^ + 2*F*_c_^2^)/3
*S* = 1.02	(Δ/σ)_max_ = 0.001
5907 reflections	Δρ_max_ = 0.14 e Å^−^^3^
433 parameters	Δρ_min_ = −0.19 e Å^−^^3^
1 restraint	Absolute structure: Flack (1983), 2608 Friedel pairs
Primary atom site location: structure-invariant direct methods	Flack parameter: 0.2 (12)

### Special details

**Table:**

Geometry. Bond distances, angles etc. have been calculated using the rounded fractional coordinates. All su's are estimated from the variances of the (full) variance-covariance matrix. The cell esds are taken into account in the estimation of distances, angles and torsion angles
Refinement. Refinement of *F*^2^ against ALL reflections. The weighted *R*-factor *wR* and goodness of fit *S* are based on *F*^2^, conventional *R*-factors *R* are based on *F*, with *F* set to zero for negative *F*^2^. The threshold expression of *F*^2^ > σ(*F*^2^) is used only for calculating *R*-factors(gt) *etc*. and is not relevant to the choice of reflections for refinement. *R*-factors based on *F*^2^ are statistically about twice as large as those based on *F*, and *R*- factors based on ALL data will be even larger.

### Fractional atomic coordinates and isotropic or equivalent isotropic displacement parameters (Å^2^)

**Table:**

	*x*	*y*	*z*	*U*_iso_*/*U*_eq_	
O1	0.1927 (3)	1.08135 (14)	0.28132 (14)	0.0610 (7)	
O2	0.0403 (3)	0.73412 (13)	0.26399 (14)	0.0597 (7)	
O3	0.1942 (3)	0.94247 (12)	0.05260 (14)	0.0620 (7)	
N1	0.1344 (3)	0.92084 (13)	0.24519 (16)	0.0473 (8)	
N2	0.1193 (3)	0.85427 (17)	0.17763 (17)	0.0458 (8)	
C1	0.1297 (3)	0.98047 (18)	0.40655 (19)	0.0429 (8)	
C2	0.1675 (4)	1.0640 (2)	0.3758 (2)	0.0458 (9)	
C3	0.1821 (4)	1.1335 (2)	0.4416 (2)	0.0515 (11)	
C4	0.1629 (4)	1.12177 (19)	0.5388 (2)	0.0524 (10)	
C5	0.1157 (4)	1.0274 (2)	0.6779 (2)	0.0588 (10)	
C6	0.0851 (5)	0.9482 (2)	0.7147 (2)	0.0718 (13)	
C7	0.0660 (5)	0.8776 (2)	0.6500 (2)	0.0699 (11)	
C8	0.0786 (4)	0.88593 (19)	0.5504 (2)	0.0573 (9)	
C9	0.1118 (3)	0.96774 (18)	0.50983 (19)	0.0434 (8)	
C10	0.1299 (4)	1.0395 (2)	0.5766 (2)	0.0471 (10)	
C11	0.1131 (4)	0.9094 (2)	0.33675 (19)	0.0443 (9)	
C12	0.0763 (3)	0.76652 (17)	0.1887 (2)	0.0468 (8)	
C13	0.1526 (3)	0.87223 (18)	0.07966 (19)	0.0453 (8)	
C14	0.1294 (3)	0.78991 (18)	0.0249 (2)	0.0446 (9)	
C15	0.0850 (4)	0.7274 (2)	0.0903 (2)	0.0451 (9)	
C16	0.0560 (4)	0.6426 (2)	0.0581 (2)	0.0560 (11)	
C17	0.0706 (4)	0.6244 (2)	−0.0405 (2)	0.0624 (11)	
C18	0.1145 (4)	0.6860 (2)	−0.1048 (2)	0.0606 (11)	
C19	0.1445 (4)	0.7711 (2)	−0.0721 (2)	0.0502 (10)	
O4	0.5655 (3)	0.70099 (13)	0.22569 (13)	0.0604 (7)	
O5	0.5765 (3)	0.84240 (12)	0.45454 (14)	0.0671 (8)	
O6	0.6863 (3)	1.05028 (13)	0.23145 (14)	0.0636 (8)	
N3	0.6153 (3)	0.86251 (12)	0.25785 (15)	0.0453 (7)	
N4	0.6275 (3)	0.93017 (16)	0.32441 (16)	0.0445 (8)	
C20	0.6151 (3)	0.80171 (17)	0.09706 (18)	0.0405 (8)	
C21	0.5877 (4)	0.7193 (2)	0.13030 (19)	0.0463 (9)	
C22	0.5778 (4)	0.6479 (2)	0.0648 (2)	0.0531 (11)	
C23	0.5919 (4)	0.65916 (19)	−0.0325 (2)	0.0528 (10)	
C24	0.6215 (4)	0.7534 (2)	−0.1750 (2)	0.0622 (11)	
C25	0.6385 (5)	0.8332 (2)	−0.2133 (2)	0.0768 (13)	
C26	0.6504 (5)	0.9047 (2)	−0.1502 (2)	0.0741 (13)	
C27	0.6458 (4)	0.89602 (18)	−0.0499 (2)	0.0586 (10)	
C28	0.6263 (3)	0.81455 (19)	−0.00696 (19)	0.0433 (9)	
C29	0.6146 (4)	0.7425 (2)	−0.0723 (2)	0.0467 (10)	
C30	0.6276 (4)	0.8741 (2)	0.16541 (19)	0.0452 (9)	
C31	0.6607 (3)	1.01881 (17)	0.30956 (19)	0.0452 (8)	
C32	0.6049 (3)	0.91258 (18)	0.42416 (18)	0.0461 (8)	
C33	0.6265 (4)	0.99628 (18)	0.4763 (2)	0.0436 (9)	
C34	0.6154 (5)	1.0177 (2)	0.5741 (2)	0.0546 (10)	
C35	0.6407 (4)	1.1019 (2)	0.6024 (2)	0.0622 (11)	
C36	0.6709 (4)	1.1646 (2)	0.5349 (2)	0.0606 (11)	
C37	0.6803 (4)	1.1441 (2)	0.4362 (2)	0.0535 (11)	
C38	0.6587 (3)	1.0595 (2)	0.40803 (19)	0.0424 (8)	
H1	0.18180	1.03670	0.24790	0.0920*	
H3	0.20520	1.18840	0.41890	0.0620*	
H4	0.17180	1.16920	0.58180	0.0630*	
H5	0.12740	1.07470	0.72120	0.0710*	
H6	0.07710	0.94120	0.78240	0.0860*	
H7	0.04420	0.82340	0.67520	0.0840*	
H8	0.06530	0.83750	0.50880	0.0690*	
H11	0.08670	0.85450	0.35830	0.0530*	
H16	0.02790	0.59970	0.10130	0.0670*	
H17	0.04980	0.56820	−0.06390	0.0750*	
H18	0.12440	0.67110	−0.17070	0.0730*	
H19	0.17400	0.81370	−0.11540	0.0600*	
H4A	0.61750	0.73840	0.26440	0.0910*	
H22	0.56140	0.59270	0.08900	0.0640*	
H23	0.58680	0.61140	−0.07470	0.0630*	
H24	0.61430	0.70550	−0.21720	0.0750*	
H25	0.64230	0.84000	−0.28160	0.0920*	
H26	0.66160	0.95940	−0.17690	0.0890*	
H27	0.65580	0.94480	−0.00910	0.0700*	
H30	0.64470	0.92940	0.14200	0.0540*	
H34	0.59120	0.97600	0.61970	0.0650*	
H35	0.63760	1.11730	0.66890	0.0750*	
H36	0.68510	1.22170	0.55600	0.0730*	
H37	0.70080	1.18640	0.39030	0.0640*	

### Atomic displacement parameters (Å^2^)

**Table:**

	*U*^11^	*U*^22^	*U*^33^	*U*^12^	*U*^13^	*U*^23^
O1	0.0772 (13)	0.0557 (13)	0.0489 (11)	−0.0106 (10)	0.0088 (9)	0.0065 (9)
O2	0.0760 (13)	0.0537 (12)	0.0485 (10)	−0.0071 (10)	0.0096 (9)	0.0120 (10)
O3	0.0853 (14)	0.0447 (11)	0.0606 (11)	−0.0025 (9)	0.0257 (10)	0.0050 (9)
N1	0.0519 (13)	0.0472 (14)	0.0428 (12)	−0.0029 (11)	0.0088 (9)	−0.0028 (11)
N2	0.0524 (13)	0.0449 (15)	0.0408 (12)	−0.0026 (10)	0.0109 (10)	0.0001 (10)
C1	0.0357 (13)	0.0446 (15)	0.0467 (15)	0.0003 (11)	0.0031 (10)	0.0005 (12)
C2	0.0401 (15)	0.0473 (17)	0.0482 (15)	−0.0023 (12)	0.0033 (11)	0.0082 (14)
C3	0.0523 (19)	0.0413 (18)	0.0598 (19)	−0.0028 (13)	0.0074 (13)	0.0039 (15)
C4	0.0492 (16)	0.0472 (17)	0.0583 (17)	−0.0015 (13)	0.0035 (12)	−0.0116 (14)
C5	0.0657 (19)	0.0622 (19)	0.0481 (16)	0.0054 (14)	0.0094 (13)	−0.0081 (13)
C6	0.102 (3)	0.070 (2)	0.0469 (16)	0.0097 (18)	0.0233 (15)	0.0064 (16)
C7	0.096 (2)	0.0573 (18)	0.0596 (17)	0.0049 (17)	0.0228 (15)	0.0109 (15)
C8	0.0718 (18)	0.0495 (16)	0.0521 (15)	0.0011 (13)	0.0155 (13)	0.0024 (12)
C9	0.0406 (14)	0.0456 (16)	0.0427 (14)	0.0036 (12)	0.0047 (10)	−0.0021 (13)
C10	0.0386 (15)	0.0509 (19)	0.0505 (16)	0.0036 (12)	0.0052 (12)	−0.0014 (14)
C11	0.0445 (14)	0.0441 (16)	0.0439 (15)	−0.0007 (12)	0.0072 (11)	−0.0003 (12)
C12	0.0407 (14)	0.0442 (15)	0.0534 (15)	−0.0003 (11)	0.0032 (11)	0.0077 (13)
C13	0.0460 (14)	0.0451 (15)	0.0454 (14)	0.0039 (12)	0.0101 (11)	0.0042 (12)
C14	0.0370 (14)	0.0465 (17)	0.0495 (17)	0.0040 (11)	0.0059 (11)	−0.0011 (13)
C15	0.0389 (14)	0.0445 (16)	0.0504 (15)	0.0012 (12)	0.0043 (11)	0.0027 (14)
C16	0.0472 (19)	0.046 (2)	0.073 (2)	−0.0024 (13)	0.0064 (14)	−0.0026 (16)
C17	0.0553 (18)	0.0529 (19)	0.076 (2)	0.0008 (15)	0.0049 (14)	−0.0188 (17)
C18	0.0573 (18)	0.069 (2)	0.0553 (17)	0.0076 (15)	0.0102 (13)	−0.0134 (16)
C19	0.0509 (16)	0.0525 (19)	0.0479 (16)	0.0085 (13)	0.0108 (12)	−0.0002 (13)
O4	0.0766 (14)	0.0559 (13)	0.0485 (11)	−0.0123 (10)	0.0110 (9)	0.0107 (10)
O5	0.1025 (16)	0.0481 (12)	0.0534 (11)	−0.0120 (11)	0.0212 (10)	0.0064 (9)
O6	0.0879 (15)	0.0543 (13)	0.0501 (11)	−0.0116 (11)	0.0167 (10)	0.0097 (10)
N3	0.0517 (12)	0.0437 (13)	0.0398 (11)	−0.0019 (11)	0.0067 (9)	−0.0048 (10)
N4	0.0556 (13)	0.0393 (14)	0.0394 (12)	−0.0034 (10)	0.0112 (10)	−0.0006 (10)
C20	0.0373 (13)	0.0430 (15)	0.0400 (13)	−0.0003 (10)	0.0038 (10)	0.0001 (11)
C21	0.0402 (15)	0.0518 (17)	0.0457 (15)	−0.0046 (12)	0.0051 (11)	0.0013 (15)
C22	0.055 (2)	0.0386 (18)	0.063 (2)	−0.0065 (13)	0.0043 (14)	−0.0001 (15)
C23	0.0503 (16)	0.0472 (18)	0.0584 (17)	−0.0020 (13)	0.0037 (12)	−0.0122 (15)
C24	0.072 (2)	0.067 (2)	0.0480 (16)	0.0105 (15)	0.0123 (13)	−0.0075 (14)
C25	0.110 (3)	0.081 (2)	0.0436 (16)	0.018 (2)	0.0255 (16)	0.0034 (17)
C26	0.113 (3)	0.0608 (19)	0.0530 (17)	0.0070 (18)	0.0271 (16)	0.0134 (15)
C27	0.082 (2)	0.0460 (17)	0.0500 (15)	0.0030 (14)	0.0179 (13)	0.0011 (12)
C28	0.0375 (14)	0.0477 (16)	0.0441 (15)	0.0012 (11)	0.0064 (10)	0.0006 (13)
C29	0.0410 (15)	0.055 (2)	0.0431 (15)	0.0030 (13)	0.0056 (11)	−0.0065 (14)
C30	0.0479 (15)	0.0454 (16)	0.0412 (14)	−0.0008 (12)	0.0054 (11)	0.0017 (12)
C31	0.0448 (15)	0.0439 (15)	0.0465 (14)	−0.0003 (11)	0.0076 (11)	0.0072 (13)
C32	0.0495 (14)	0.0465 (16)	0.0421 (14)	−0.0033 (12)	0.0080 (11)	0.0035 (12)
C33	0.0380 (14)	0.0453 (17)	0.0469 (16)	0.0036 (11)	0.0065 (11)	−0.0024 (12)
C34	0.0529 (17)	0.069 (2)	0.0433 (16)	0.0020 (14)	0.0128 (12)	−0.0029 (14)
C35	0.0544 (17)	0.073 (2)	0.0585 (18)	0.0048 (15)	0.0088 (13)	−0.0235 (17)
C36	0.0568 (18)	0.0501 (19)	0.073 (2)	0.0033 (15)	0.0073 (14)	−0.0154 (17)
C37	0.0496 (19)	0.0461 (19)	0.0627 (19)	−0.0025 (13)	0.0049 (13)	−0.0001 (15)
C38	0.0359 (13)	0.0442 (15)	0.0462 (14)	0.0007 (11)	0.0055 (10)	−0.0022 (13)

### Geometric parameters (Å, °)

**Table:**

O1—C2	1.343 (3)	C6—H6	0.9300
O2—C12	1.200 (3)	C7—H7	0.9300
O3—C13	1.203 (3)	C8—H8	0.9300
O1—H1	0.8200	C11—H11	0.9300
O4—C21	1.352 (3)	C16—H16	0.9300
O5—C32	1.193 (3)	C17—H17	0.9300
O6—C31	1.203 (3)	C18—H18	0.9300
O4—H4A	0.8200	C19—H19	0.9300
N1—C11	1.281 (3)	C20—C21	1.380 (4)
N1—N2	1.366 (3)	C20—C28	1.430 (3)
N2—C12	1.409 (4)	C20—C30	1.443 (4)
N2—C13	1.410 (3)	C21—C22	1.408 (4)
N3—N4	1.371 (3)	C22—C23	1.342 (4)
N3—C30	1.275 (3)	C23—C29	1.419 (4)
N4—C32	1.407 (3)	C24—C29	1.401 (4)
N4—C31	1.415 (4)	C24—C25	1.354 (4)
C1—C2	1.401 (4)	C25—C26	1.389 (4)
C1—C11	1.438 (4)	C26—C27	1.362 (4)
C1—C9	1.432 (4)	C27—C28	1.407 (4)
C2—C3	1.386 (4)	C28—C29	1.414 (4)
C3—C4	1.352 (4)	C31—C38	1.469 (4)
C4—C10	1.409 (4)	C32—C33	1.469 (4)
C5—C6	1.357 (4)	C33—C38	1.391 (4)
C5—C10	1.397 (4)	C33—C34	1.373 (4)
C6—C7	1.389 (4)	C34—C35	1.362 (4)
C7—C8	1.365 (4)	C35—C36	1.374 (4)
C8—C9	1.418 (4)	C36—C37	1.378 (4)
C9—C10	1.420 (4)	C37—C38	1.366 (4)
C12—C15	1.467 (4)	C22—H22	0.9300
C13—C14	1.467 (4)	C23—H23	0.9300
C14—C15	1.385 (4)	C24—H24	0.9300
C14—C19	1.360 (4)	C25—H25	0.9300
C15—C16	1.388 (4)	C26—H26	0.9300
C16—C17	1.378 (4)	C27—H27	0.9300
C17—C18	1.364 (4)	C30—H30	0.9300
C18—C19	1.395 (4)	C34—H34	0.9300
C3—H3	0.9300	C35—H35	0.9300
C4—H4	0.9300	C36—H36	0.9300
C5—H5	0.9300	C37—H37	0.9300
			
C2—O1—H1	109.00	C16—C17—H17	119.00
C21—O4—H4A	110.00	C19—C18—H18	120.00
N2—N1—C11	121.7 (2)	C17—C18—H18	120.00
N1—N2—C13	117.8 (2)	C14—C19—H19	121.00
C12—N2—C13	111.6 (2)	C18—C19—H19	121.00
N1—N2—C12	130.6 (2)	C21—C20—C28	118.9 (2)
N4—N3—C30	121.4 (2)	C28—C20—C30	120.5 (2)
C31—N4—C32	112.0 (2)	C21—C20—C30	120.6 (2)
N3—N4—C32	118.0 (2)	O4—C21—C20	123.4 (2)
N3—N4—C31	130.0 (2)	C20—C21—C22	121.3 (2)
C9—C1—C11	121.1 (2)	O4—C21—C22	115.3 (3)
C2—C1—C11	120.8 (2)	C21—C22—C23	120.2 (3)
C2—C1—C9	118.1 (2)	C22—C23—C29	121.4 (3)
C1—C2—C3	121.7 (2)	C25—C24—C29	120.5 (3)
O1—C2—C3	116.2 (3)	C24—C25—C26	119.8 (3)
O1—C2—C1	122.1 (3)	C25—C26—C27	121.1 (3)
C2—C3—C4	120.2 (3)	C26—C27—C28	121.2 (3)
C3—C4—C10	121.6 (3)	C20—C28—C27	123.7 (3)
C6—C5—C10	121.5 (3)	C27—C28—C29	117.0 (2)
C5—C6—C7	119.2 (3)	C20—C28—C29	119.4 (3)
C6—C7—C8	121.5 (3)	C23—C29—C24	120.6 (3)
C7—C8—C9	120.6 (3)	C23—C29—C28	118.9 (2)
C1—C9—C10	119.4 (2)	C24—C29—C28	120.6 (3)
C8—C9—C10	117.4 (2)	N3—C30—C20	120.3 (3)
C1—C9—C8	123.2 (2)	O6—C31—N4	125.0 (2)
C5—C10—C9	119.8 (3)	N4—C31—C38	105.1 (2)
C4—C10—C9	118.9 (2)	O6—C31—C38	129.9 (3)
C4—C10—C5	121.4 (3)	O5—C32—C33	130.7 (2)
N1—C11—C1	120.7 (3)	N4—C32—C33	105.3 (2)
N2—C12—C15	104.9 (2)	O5—C32—N4	124.1 (2)
O2—C12—C15	129.8 (3)	C32—C33—C38	108.8 (2)
O2—C12—N2	125.4 (2)	C34—C33—C38	120.5 (3)
N2—C13—C14	105.9 (2)	C32—C33—C34	130.6 (3)
O3—C13—N2	123.8 (2)	C33—C34—C35	118.1 (3)
O3—C13—C14	130.3 (2)	C34—C35—C36	121.5 (3)
C13—C14—C15	107.9 (2)	C35—C36—C37	121.0 (3)
C13—C14—C19	130.2 (3)	C36—C37—C38	117.7 (3)
C15—C14—C19	121.8 (3)	C31—C38—C37	130.0 (3)
C14—C15—C16	120.2 (2)	C33—C38—C37	121.2 (2)
C12—C15—C16	130.1 (3)	C31—C38—C33	108.8 (3)
C12—C15—C14	109.7 (3)	C21—C22—H22	120.00
C15—C16—C17	117.4 (3)	C23—C22—H22	120.00
C16—C17—C18	122.3 (3)	C22—C23—H23	119.00
C17—C18—C19	120.2 (3)	C29—C23—H23	119.00
C14—C19—C18	118.0 (3)	C25—C24—H24	120.00
C4—C3—H3	120.00	C29—C24—H24	120.00
C2—C3—H3	120.00	C24—C25—H25	120.00
C10—C4—H4	119.00	C26—C25—H25	120.00
C3—C4—H4	119.00	C25—C26—H26	119.00
C10—C5—H5	119.00	C27—C26—H26	119.00
C6—C5—H5	119.00	C26—C27—H27	119.00
C5—C6—H6	120.00	C28—C27—H27	119.00
C7—C6—H6	120.00	N3—C30—H30	120.00
C8—C7—H7	119.00	C20—C30—H30	120.00
C6—C7—H7	119.00	C33—C34—H34	121.00
C9—C8—H8	120.00	C35—C34—H34	121.00
C7—C8—H8	120.00	C34—C35—H35	119.00
N1—C11—H11	120.00	C36—C35—H35	119.00
C1—C11—H11	120.00	C35—C36—H36	120.00
C15—C16—H16	121.00	C37—C36—H36	119.00
C17—C16—H16	121.00	C36—C37—H37	121.00
C18—C17—H17	119.00	C38—C37—H37	121.00
			
C11—N1—N2—C12	1.8 (4)	C13—C14—C15—C16	−179.7 (2)
C11—N1—N2—C13	−177.5 (2)	C13—C14—C15—C12	0.4 (3)
N2—N1—C11—C1	180.0 (2)	C13—C14—C19—C18	−179.9 (2)
C13—N2—C12—C15	0.4 (3)	C19—C14—C15—C12	−179.3 (2)
C12—N2—C13—O3	−179.0 (2)	C19—C14—C15—C16	0.6 (4)
N1—N2—C13—C14	179.3 (2)	C15—C14—C19—C18	−0.2 (4)
C12—N2—C13—C14	−0.1 (3)	C14—C15—C16—C17	−0.9 (4)
N1—N2—C13—O3	0.4 (4)	C12—C15—C16—C17	178.9 (3)
N1—N2—C12—O2	1.5 (4)	C15—C16—C17—C18	1.0 (4)
C13—N2—C12—O2	−179.3 (2)	C16—C17—C18—C19	−0.7 (5)
N1—N2—C12—C15	−178.9 (2)	C17—C18—C19—C14	0.3 (4)
C30—N3—N4—C31	−2.6 (4)	C30—C20—C21—C22	−179.1 (3)
C30—N3—N4—C32	177.5 (2)	C28—C20—C21—C22	2.4 (4)
N4—N3—C30—C20	179.7 (2)	C30—C20—C21—O4	2.0 (4)
C32—N4—C31—C38	0.0 (3)	C28—C20—C30—N3	179.2 (2)
C31—N4—C32—O5	−179.6 (2)	C30—C20—C28—C29	179.7 (2)
N3—N4—C32—C33	179.6 (2)	C21—C20—C30—N3	0.7 (4)
C31—N4—C32—C33	−0.3 (3)	C28—C20—C21—O4	−176.5 (2)
N3—N4—C32—O5	0.3 (4)	C30—C20—C28—C27	−1.3 (4)
N3—N4—C31—O6	−0.6 (4)	C21—C20—C28—C27	177.2 (2)
C32—N4—C31—O6	179.3 (2)	C21—C20—C28—C29	−1.8 (3)
N3—N4—C31—C38	−179.9 (2)	O4—C21—C22—C23	177.9 (3)
C9—C1—C2—O1	177.8 (2)	C20—C21—C22—C23	−1.1 (4)
C9—C1—C11—N1	−178.1 (2)	C21—C22—C23—C29	−0.8 (4)
C11—C1—C2—C3	179.3 (3)	C22—C23—C29—C28	1.3 (4)
C11—C1—C2—O1	−1.1 (4)	C22—C23—C29—C24	−177.5 (3)
C9—C1—C2—C3	−1.9 (4)	C25—C24—C29—C28	−0.3 (4)
C2—C1—C9—C8	−178.3 (2)	C29—C24—C25—C26	0.4 (5)
C2—C1—C9—C10	0.9 (3)	C25—C24—C29—C23	178.4 (3)
C2—C1—C11—N1	0.7 (4)	C24—C25—C26—C27	0.2 (5)
C11—C1—C9—C10	179.7 (2)	C25—C26—C27—C28	−0.9 (5)
C11—C1—C9—C8	0.5 (4)	C26—C27—C28—C29	0.9 (4)
C1—C2—C3—C4	1.1 (4)	C26—C27—C28—C20	−178.1 (3)
O1—C2—C3—C4	−178.6 (3)	C27—C28—C29—C24	−0.3 (4)
C2—C3—C4—C10	0.7 (4)	C20—C28—C29—C24	178.8 (2)
C3—C4—C10—C9	−1.6 (4)	C27—C28—C29—C23	−179.1 (3)
C3—C4—C10—C5	178.1 (3)	C20—C28—C29—C23	0.0 (4)
C6—C5—C10—C9	0.1 (4)	O6—C31—C38—C37	2.0 (4)
C10—C5—C6—C7	−0.5 (5)	N4—C31—C38—C33	0.3 (3)
C6—C5—C10—C4	−179.6 (3)	N4—C31—C38—C37	−178.8 (3)
C5—C6—C7—C8	0.5 (5)	O6—C31—C38—C33	−178.9 (3)
C6—C7—C8—C9	0.0 (5)	O5—C32—C33—C34	−1.8 (5)
C7—C8—C9—C10	−0.4 (4)	O5—C32—C33—C38	179.7 (3)
C7—C8—C9—C1	178.9 (3)	N4—C32—C33—C34	178.9 (3)
C8—C9—C10—C4	−180.0 (3)	N4—C32—C33—C38	0.5 (3)
C1—C9—C10—C5	−179.0 (2)	C32—C33—C34—C35	−179.6 (3)
C1—C9—C10—C4	0.7 (4)	C38—C33—C34—C35	−1.3 (5)
C8—C9—C10—C5	0.3 (4)	C32—C33—C38—C31	−0.5 (3)
N2—C12—C15—C16	179.7 (3)	C32—C33—C38—C37	178.7 (2)
N2—C12—C15—C14	−0.5 (3)	C34—C33—C38—C31	−179.1 (3)
O2—C12—C15—C14	179.1 (3)	C34—C33—C38—C37	0.1 (4)
O2—C12—C15—C16	−0.7 (5)	C33—C34—C35—C36	1.9 (5)
N2—C13—C14—C19	179.5 (3)	C34—C35—C36—C37	−1.3 (5)
O3—C13—C14—C15	178.6 (3)	C35—C36—C37—C38	0.0 (4)
N2—C13—C14—C15	−0.2 (3)	C36—C37—C38—C31	179.6 (2)
O3—C13—C14—C19	−1.7 (5)	C36—C37—C38—C33	0.6 (4)

### Hydrogen-bond geometry (Å, °)

**Table:**

*D*—H···*A*	*D*—H	H···*A*	*D*···*A*	*D*—H···*A*
O1—H1···N1	0.82	1.83	2.555 (3)	147
O4—H4A···N3	0.82	1.93	2.555 (3)	133
C18—H18···O6^i^	0.93	2.53	3.204 (4)	129
C35—H35···O4^ii^	0.93	2.56	3.339 (4)	141

Symmetry codes: (i) −*x*+1, *y*−1/2, −*z*; (ii) −*x*+1, *y*+1/2, −*z*+1.

### Table 2 π–π interactions (Å)

**Table:**

π–π	Distance
Cg2···Cg4	3.5627 (19)
Cg4···Cg6	3.5527 (19)
Cg1···Cg6^i^	3.7758 (19)
Cg2···Cg5^i^	3.7759 (19)
Cg2···Cg8^i^	3.693 (2)
Cg3···Cg8^i^	3.784 (2)
Cg4···Cg6^i^	3.7065 (19)
Cg4···Cg7^i^	3.831 (2)

Cg1 = Centroid of ring [N2,C12-C15], Cg2 = Centroid of ring [C1-C4,C9,C10]; Cg3 = Centroid of ring [C5-C10]; Cg4 = centroid of ring [C14-C19]; Cg5 = Centroid of ring [N4,C31-C33,C38]; Cg6 = centroid of ring [C20-C23,C28,C29]; Cg7 = Centroid of ring [C24-C29]; Cg8 = centroid of ring [C33-C38]; Symmetry operator: (i) x-1, y, z.
